# Long-haul and high-resolution optical time domain reflectometry using superconducting nanowire single-photon detectors

**DOI:** 10.1038/srep10441

**Published:** 2015-05-28

**Authors:** Qingyuan Zhao, Lan Xia, Chao Wan, Junhui Hu, Tao Jia, Min Gu, Labao Zhang, Lin Kang, Jian Chen, Xuping Zhang, Peiheng Wu

**Affiliations:** 1 Research Institute of Superconductor Electronics (RISE), School of Electronic Science and Engineering, Nanjing University, 22 Hankou Road, Nanjing 210093, China; 2 Institute of Optical Communication Engineering, School of Management and Engineering, Nanjing University, 22 Hankou Road, Nanjing, 210093, China; 3 College of Physics Science and Technology, Guangxi Normal University, 15 Yucai Road, Guilin, 541004, China

## Abstract

In classical optical time domain reflectometries (OTDRs), for sensing an 200-km-long fiber, the optical pulses launched are as wide as tens of microseconds to get enough signal-to-noise ratio, while it results in a two-point resolution of kilometers. To both reach long sensing distance and sub-kilometer resolution, we demonstrated a long-haul photon-counting OTDR using a superconducting nanowire single-photon detector. In a 40-minute-long measurement, we obtained a dynamic range of 46.9 dB, corresponding to a maximum sensing distance of 246.8 km, at a two-point resolution of 0.1 km. The time for measuring fiber after 100 km was reduced to one minute, while the fiber end at 217 km was still distinguished well from noise. After reducing the pulse width to 100 ns, the experimental two-point resolution was improved to 20 m while the maximum sensing distance was 209.47 km.

Optical time domain reflectometry (OTDR) is an essential tool for characterizing long fibers^1^. It can nondestructively detect fiber properties, such as length, attenuation, and locations of connectors, splices or faults over hundreds of kilometers. The backscattered light is detected at the same site where optical pulses are launched, facilitating measurements of a long fiber with its one terminator buried in the ground or under the sea. In OTDRs, there is a tradeoff between the maximal distance range and two-point resolution. For example, in a commercial OTDR (FTB-7600E ultra-long-haul OTDR from EXFO), to reach a two-point resolution of 0.1 km (optical pulse width is 1 μs), the maximum distance range is only 80 km. By launching 10 μs wide optical pulses, the distance range increases to 260 km, but the two-point resolution deteriorates to 1 km. Moreover, to get a clear OTDR trace, the measurement will take hours to average signals. Photon-counting OTDRs, also called ν-OTDRs, use single-photon detectors instead of linear photon diodes in classical OTDRs^2^. Because of the ultra-sensitive single-photon detectors and the photon-counting technology, ν-OTDRs can have larger dynamic range^3^, better two-point resolution^4^, and classical dead-zone avoidance^5^. Therefore, longer fiber links can be diagnosed by a ν-OTDR at a better two-point resolution.

ν-OTDRs have been demonstrated in different single-photon detectors. Geiger-mode-operated InGaAs/InP avalanche photodiodes (APDs) are most commonly used due to their flexibility and robustness^2^. In order to reduce dark counts while maintaining a high efficiency at telecom wavelengths, a quenching circuit turns the diode off for microseconds once it is triggered. Consequently, it leads to a dead time during which gated APDs are completely unable to detect photons. In other words, it takes a longer sampling time for compensating the signal loss of backscattered light during the dead time. Moreover, afterpulses and charge persistence effects in APDs can create a dead zone after a large loss event or a strong reflection, degrading the two-point resolution^6^. Optical frequency up-conversion is another single-photon detection technique, on the principle of using a free-running and high efficiency silicon APD to detect visible light that is converted from near-infrared light in a non-linear optical medium^3^,^7^. To filter off nonlinear noise during the up-conversion process, the pump laser and pulsed laser are in narrow bandwidths. Nevertheless, light pulses with narrow bandwidth generate coherent noise in OTDR traces^6^. Moreover, up-conversion detectors are usually polarization-dependent, adding more fluctuation in OTDR traces^8^.

Among several kinds of single-photon detectors that have been implemented in ν-OTDRs, superconducting nanowire single-photon detectors (SNSPDs) have shown superior performance in high efficiency, low dark counts, and low timing jitter^9^,^10^,^11^, which govern the distance and two-point resolution in OTDRs. SNSPDs operate in a free-running mode without quenching or other synchronized circuits. Thus, the detector counts backscattered photons continuously, increasing the signal-to-noise ratio of the OTDR trace and reducing the total measurement time. When the detector is biased appropriately, there are no afterpulses or charge-persistence-like effects. Therefore, the detector’s timing jitter is the primary factor that gives additional spread to the two-point resolution^4^. For SNSPDs, the timing jitter is typically in several tens of picoseconds, corresponding to a spread of several millimeters, and can be ignored in long-haul OTDRs.

We implemented three main improvements to extend the dynamic range of an OTDR based on our previous work^12^. Since the intensity of backscattered signal in a photon counting ν-OTDR is represented by statistically summing counts in every time bin, both increasing signal counts (the upper boundary of a dynamic range) and reducing noise floor (the lower boundary of a dynamic range) within a measurement time can increase the dynamic range and thus raise the sensing distance. First, we integrated an optic cavity on the SNSPD to reach a high system detection efficiency to increase signal counts^13^. Secondly, we designed a non-latch circuit for reading SNSPD pulses at strong backscattered light^14^. Thus, the SNSPD operated in a robust free-running mode, collecting all the backscattered light in one period. Thirdly, the laser in this work was modulated internally for outputting a clear pulse with an extinction ratio over 100 dB. Thus, the noise floor was reduced by removing noise from stray light. Overall, by these improvements, within the same measurement time, more single counts and less dark counts were collected, resulting in a higher dynamic range, corresponding to a longer sensing distance.

Using the upgraded OTDR system, we obtained a dynamic range of 46.9 dB which gave a maximum sensing distance of 246.8 km for a single-mode fiber of 0.19 dB/km loss, by launching 1 μs wide optical pulses and measuring for 40 minutes. The two-point resolution was 0.1 km. The measurement time for the fiber after 100 km was reduced to one minute while the dynamic range was reduced slightly to 43.1 dB. By reducing the pulse width to 100 ns and measuring for 60 minutes, including the broadening of pulse after transmitting hundreds of kilometers in fiber, the two-point resolution decreased to 20 m. Under these conditions, the dynamic range was 39.8 dB, which was still capable of detecting fiber over 200 km. In both measurements, fluctuations on the OTDR trace were only caused by the intrinsic photon-counting mechanism in which massive counts give low deviations from the average value. The detector and laser gave negligible fluctuations. Dependences of the dynamic range and fluctuation on measurement time were both analyzed to give guidance to balance the tradeoff between measurement time and OTDR specifications.

## Materials and Methods

### Experimental setup

**Figure 1:**
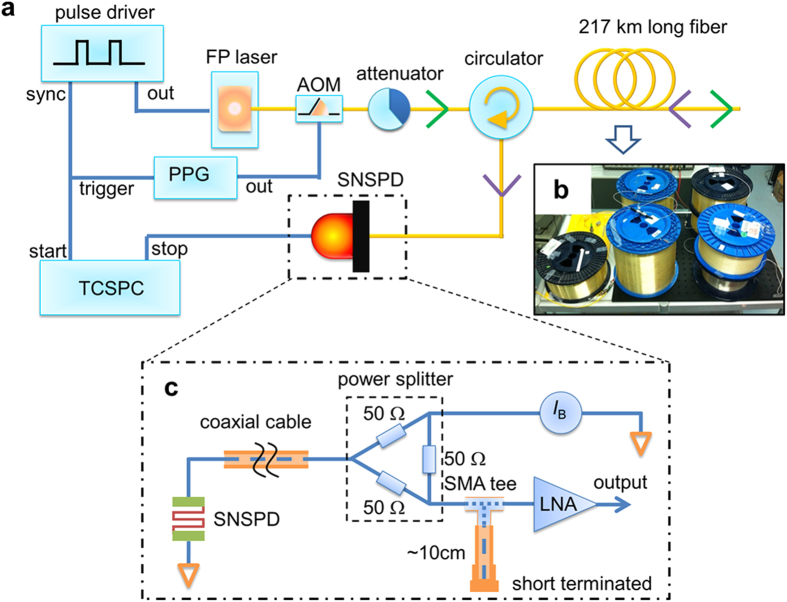
ν-OTDR setup using an superconducting nanowire single-photon detector. (**a**) Schematic setup of a ν-OTDR using an SNSPD. The attenuator was connected for adjusting the power of incident optical pulses to prevent the detector from saturating when the initial fiber was measured. (**b**) Under-tested fiber. There are two fiber spools fold together on the top right. The fiber spool sat under the blue spool was not connected. (**c**) The “capacitor-grounded” readout circuit for reading out SNSPD’s outputs.

Figure 1a is our long-haul OTDR setup. To realize a high dynamic range, pure optical pulses with high extinction ratio are necessary. To obtain these, we internally modulated a Fabry-Perot (FP) laser diode (Thorlab, FPL1009S) with a squared pulse driver. Although a reverse bias was applied on the diode to force it to be off, we still observed some leakage light. Hence, an acousto-optic modulator (AOM) controlled by a pulse pattern generator (PPG) was connected after the diode to remove the leakage light. But the AOM introduced an additional 3 dB loss. The repetition rate (*f*_r_) of the diode was 100 Hz, which was slow enough to ensure that the backscattered signals did not overlap between successive pulses. The widths of optical pulses (*t*_P_) were tuned to 1 μs and 100 ns for two independent measurements. The center wavelength of the output light was 1550 nm with a bandwidth of 10 nm, which gave negligible coherent Rayleigh noise. The 10 nm-bandwidth pulses spread in the fiber due to dispersions and thus the two-point resolution was deteriorated in the OTDR measurement with 100 ns optical pulses. However, in the OTDR measurement with 1 μs optical pulses, the pulse spread was only a small portion of the total pulse width. The peak power of the optical pulses was 6 dBm, measured by a calibrated photodiode. When measuring the initial fiber, we attenuated the input pulses or reduced the biased voltage of the diode to lower the power of the backscattered light to prevent the SNSPD from saturating. The optical pulses then entered into six fiber spools (as shown in Fig. 1b) with a total length of 217 km through a circulator. The SNSPD was operated in a liquid helium flow cryostat at a temperature of 2.0 K. Time delays between the SNSPD’s outputs and input optical pulses were collected and analyzed by a time-correlated single-photon detection card (TCSPC, HydraHarp400). The time bin size (*t*_b_) was set according to the optical pulse width.

### Superconducting nanowire single-photon detector

The SNSPD was fabricated on a thermal oxidized silicon substrate. The thickness of the thermal oxidized SiO_2_ was 270 nm on both sides. We grew another 250 nm thick SiO on top of the meander wire and then covered the whole device with a gold layer. Therefore, the SNSPD was embedded in an optical cavity which enhanced the absorptance over 90%. The SiO_2_ layer on the back reduced the reflection to increase the fiber coupling efficiency. Detectors with similar cavity structures were reported for high system efficiencies by other groups^15^,^16^,^17^. The patterned NbN nanowire had a width of 100 nm and a pitch of 200 nm over an active area of 15 × 15 μm^2^. The system detection efficiency, including coupling loss and fiber loss in the dip-stick, was 10% at a dark count of 1.7 Hz at a temperature of 2.0 K, resulting in *NEP*_D_  = 2.4 × 10^−18^ W/√Hz. Outputs of the SNSPD were read out by the “capacitor-grounded” circuit as shown in Fig. 1c^14^. A short-terminated coaxial cable was paralleled at the input of a low noise amplifier (LNA). Therefore, any potential that was charged by frequent pulses was quickly discharged to ground. The short-terminated coaxial cable can also reset the SNSPD when it was temporarily switched to normal state by strong reflected lights or electrical noise. Thus, the SNSPD was operating in a free-running mode with a constant biased current of 0.85 of its critical current over hours.

**Figure 2:**
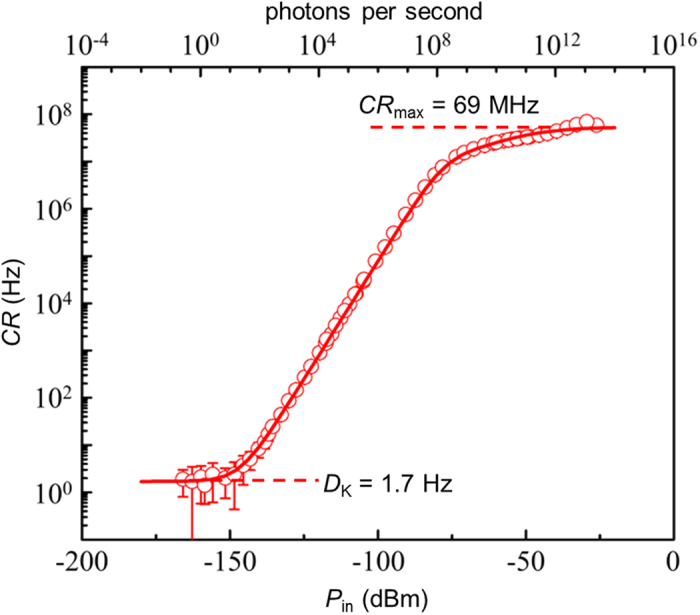
Counting rate (*CR*) versus incident light power (*P*_in_) of the SNSPD. Fitting curve (red solid line) applied on the symbols is in good agreement with the experimental data. The counting rates at the lower and upper plateaus give the dark counts and the maximum counting rates, respectively.

Since the power of backscattered signals in a long-haul ν-OTDR ranges from single-photon level to an intensive light of tens of decibels higher, we first investigated the range of light intensity that the SNSPD can respond to. In this measurement, the laser diode was biased at a constant current and thus it output continuous light. We adjusted the light power (*P*_in_) and recorded the counting rate (*CR*). The *CR* vs. *P*_in_ curve is shown in Fig. 2. We also calculated the mean photons per second (μ) and plotted it as an additional axis for reading the detection efficiency. When *P*_in_ was very weak, *CR* represented the dark counting rate (*D*_K_), which was *D*_K_ = 1.7 Hz. The slope of the linear range *η* = *CR*/μ gave the detection efficiency which was 10% for the SNSPD. Even when *P*_in_ went up to -29 dBm, the SNSPD could still count at *CR*_max_ = 69 MHz. Using a numerical model for free-running SNSPD^14^, we fitted the experimental data in Fig. 2. The fitting curves agreed well with the data and thus the same fitting method can be used to correct ν-OTDR traces where the detector saturated. We used the range in which *η* changed less than 3 dB from the detection efficiency of single-photon detections to define the linear response range. The range of *P*_in_ where the SNSPD responded linearly was from -143 dBm to -81 dBm (a total span of 62 dB), which can represent a linear dynamic range of 31 dB in one OTDR measurement.

### Two-step measurement

The measurement took two steps. In the first measurement, incident light pulses were not attenuated, thus the peak power was maximized to 3 dBm, which was so strong that the trace saturated at the initial fiber. The peak power was about one order of magnitude lower than the values reported in other long-haul OTDR measurements^3^,^18^,^19^, indicating the dynamic range of our system can be further increased by using a more powerful laser. To test the initial fiber, we attenuated the optical pulses by 40 dB to take a second measurement. In the two-step measurement, the first step took most of the measurement time, because the backscattered light from the far end of the fiber was so weak that it was counted as photons. The information from the first step was most interesting because it gave the fiber properties hundreds of kilometers far away and it was unable to be measured by conventional OTDRs. The second step could still be speeded up by merging more ν-OTDR traces with less attenuation. This two-step measurement was fundamentally more efficient than the rapid-gating measurement in OTDRs using APDs^2^, because the SNSPD always operated in free-running mode at its maximum performance until its output pulses saturated. For every measured ν-OTDR traces, we did a noise correction to remove the dark counts (see Supplementary Materials for details). The vertical axis was converted from the total photon counts *C*, to 5log(*C*) or the loss of the backscattered signal for convenience in discussions.

## Results and Discussions

### Long-haul ν-OTDR

**Figure 3:**
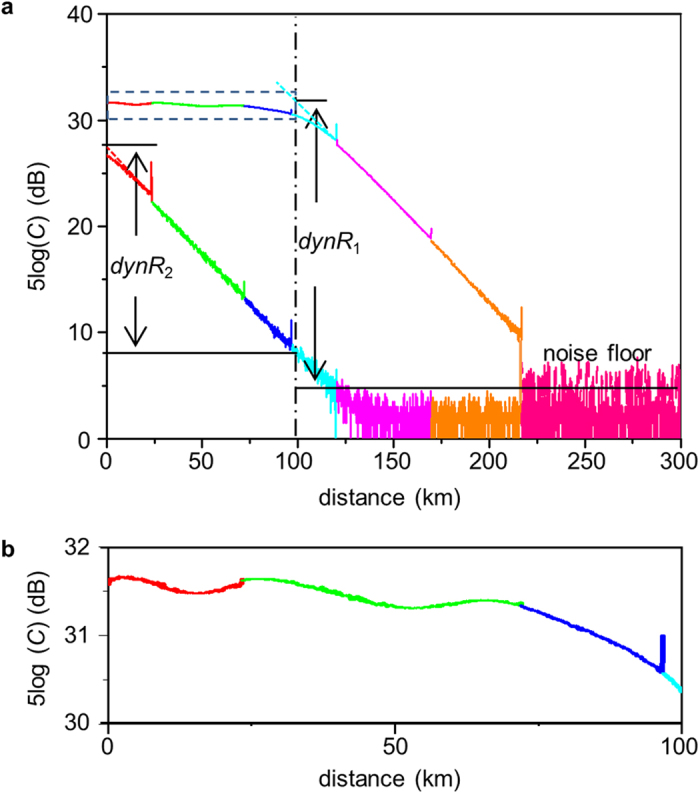
Long-haul OTDR traces. (**a**) OTDR traces of six spools of fiber with a total length of 217 km. The six fiber spools and the noise floors are marked in different colors. The trace on top is from the first 30-minute-long measurement without attenuating optical pulses while the bottom one is from the second 10-minute-long measurement after attenuating optical pulses by 40 dB. The dashed lines are linear corrections of the saturated curves. (**b**) An enlarged view of the initial 100-km-long OTDR trace in the first measurement, where reflection peaks can be seen. The oscillation of the curve comes from the current reset dynamics of SNSPDs.

The ν-OTDR traces for the 217 km long fiber spools are shown in Fig. 3a. The width of optical pulses was *t*_p_ = 1 μs and so was the time bin window. It took 30 minutes and 10 minutes for the first and the second measurement. As shown in Fig. 3b, although the optical pulses launched into the fiber in the first measurement were so strong that the trace saturated at the initial fiber, reflection peaks from the connections were visible. In other words, even though the attenuation property of the fiber was blinded, some information, such as location of connections or serious attenuations, could still be determined. To test the attenuation of the initial 100 km long fiber, we took a quick second measurement.

From this two-step measurement, we found locations for all the connections and calculated the fiber attenuation of each fiber spool, which were the basic functions of an OTDR. For calculating the ultimate dynamic range, we defined the root-mean-square (RMS) value of the noise trace in the first measurement as the noise floor, which was 5 dB. We corrected the saturated region by using a linear extrapolation with the measured fiber attenuation. Then, the dynamic range of the fiber tail from 100 km to the noise floor was *dynR*_1_ = 27.1 dB, and the dynamic range of the fiber before 100 km was *dynR*_2_ = 19.8 dB. Therefore, the total dynamic range of our OTDR system was *dynR* = *dynR*_1_ + *dynR*_2_ = 46.9 dB.

### High two-point resolution

**Figure 4:**
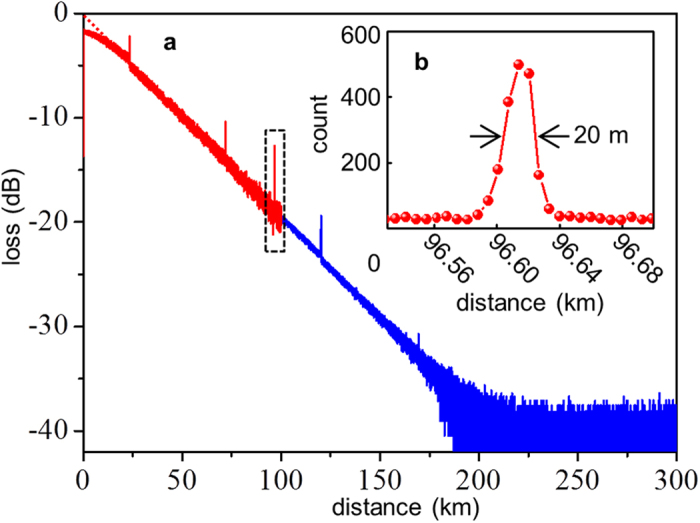
High two-point resolution OTDR traces. (**a**) OTDR trace from launching 100-ns-wide optical pulses. The overall measurements were taken from the two-step measurement. The dashed red line is from a linear correction of the saturated region at the beginning. (**b**) An enlarged view of the Fresnel reflection peak in the dashed box in (**a**) at 96.62 km. The vertical axis changes to linear scale of the total sampling count in each time bin. The FWHM of the peak gives a two-point resolution of 20 m.

Two-point resolution is defined as the minimal distance from which two reflection peaks can be distinguished. It is approximately equal to the full-width-at-half-maximum (FWHM) of a single reflection peak. To achieve a better two-point resolution, we reduced the width of optical pulse to *t*_p_ = 100 ns and set the time bin window to *t*_b_ = 66 ns. We took the two-step measurements, each of which was 30 minutes long. We merged the initial 100 km trace and the rest trace after 100 km into a single ν-OTDR trace. The vertical axis of the ν-OTDR trace was converted to the loss of the backscattered light. Fig. 4a gives the overall ν-OTDR trace. The total dynamic range was 39.8 dB, corresponding to a maximum length of 209.47 km.

We investigated the two-point resolution by picking out a Fresnel reflection peak at 96.62 km, where the reflection was the strongest. Ideally, by launching 100 ns wide optical pulses, the two-point resolution should be in a square profile with a width of *t*_P_∙*v*/2 = 10 m, where *v* = 2.0 × 10^8^ m/s was the light velocity in fiber. However, as shown in Fig. 4b, the experimental peak was distorted into a profile with slow rising and falling edges with a FWHM of 20 m. There were two factors accounting for this distortion. First, the sampling resolution was *t*_b_∙*v*/2 = 6.56 m, which was not fine enough to draw the edges precisely. Additionally, because of dispersions in fiber, a squared optical pulse spread out after a long transmission. In order to acquire a better two-point resolution, short optical pulses and fine sampling are required. However, in that case, the dynamic range decreases and the measuring time is longer. In our previous work, for a short fiber where dispersion effects were negligible, we demonstrated that the ultimate two-point resolution was 4.0 mm and was only limited by the SNSPD’s timing jitter^4^. In this paper, the detector read out by the “capacitor-grounded” circuit had a timing jitter of 87 ps, corresponding to an ultimate two-point resolution of 8.7 mm.

### Fast measurement

Because the SNSPD operated in a free-running mode, the measurement time was much shorter than the time using a gated APD or a gated SNSPD^2^,^19^. In ν-OTDRs, the dynamic range can be increased and the fluctuation can be reduced with a longer measurement time, but the slow measurements will limit the practicability. Thus, we investigated the dependence of dynamic range and fluctuation on measurement time. It was found experimentally that the measurement time could be as short as one minute to test the fiber over 200 km.

**Figure 5:**
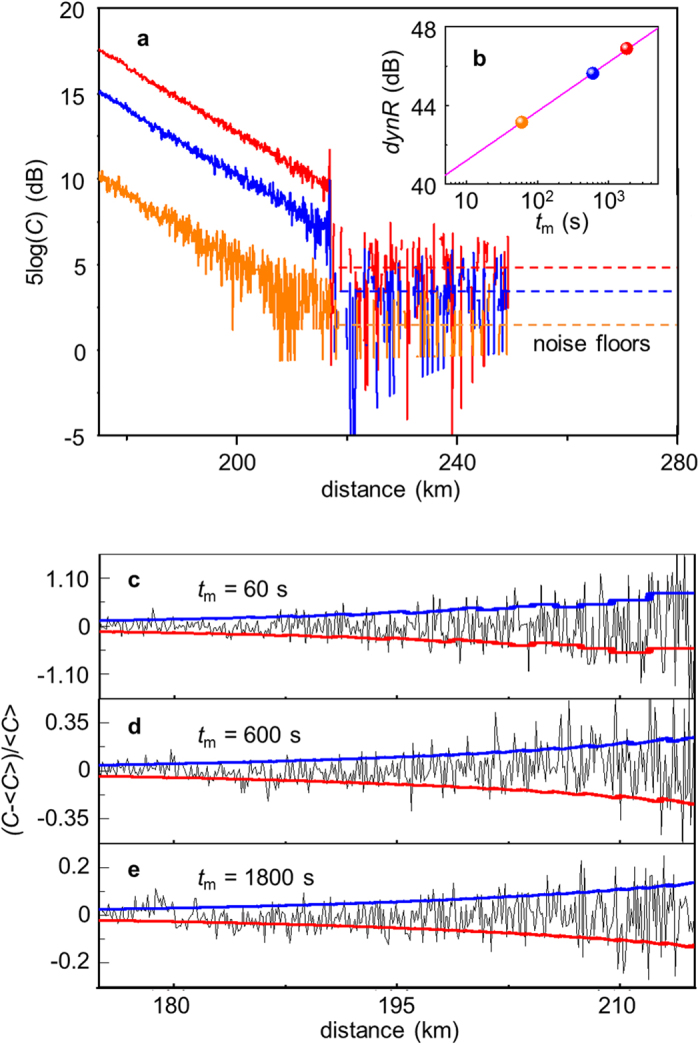
Fluctuations of OTDR traces versus measurement times. (**a**) OTDR traces from taking the first measurements 60 s (orange), 600 s (blue), and 1800 s (red). As the measurement time increases, both signal counts and noise counts go up. But, the signal counts increase faster than the noise counts, thus the dynamic range increases. (**b**) The dynamic ranges (*dynR*) versus measurement time *t*_m_. (**c**)~(e) Variations of the OTDR traces from 175 km to 215 km at different measurement times. Scales of the vertical axes are proportional to 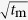. In each case, the experimental variations are well confined between the upper boundary (blue) and lower boundary (red). Both boundaries are calculated from a Poisson distribution statistics.

Since the first step for measuring the fiber tail took most of the time, and determined the noise floor as well as the total dynamic range, we only discussed the measurement time for the first step. We did the first measurements for 60 s, 600 s, and 1800 s. The obtained OTDR traces are shown in Fig. 5a. The dynamic ranges were 43.1 dB, 45.6 dB, and 46.9 dB, with noise floors of 1.2 dB, 3.7 dB, and 5.0 dB, respectively. As shown in Fig. 5b, the dynamic range follows the measurement time (in log scale) linearly, which is in good agreement with a theoretical fitting from photon statistics (see Supplementary Materials for the fitting method). Our experimental results showed that the dynamic range of a one-minute-long measurement was 43.1 dB, which was capable of testing fiber over 200 km.

In a fast measurement, the fluctuation of a ν-OTDR trace needs to be minimized so that the fiber information can be inferred accurately. In our ν-OTDR system, the bandwidth of the FP laser diode was 10 nm, which was wide enough to remove coherent speckles. In ν-OTDRs using up-conversion technologies, there was polarization noise contributing to the total fluctuation^8^. SNSPDs were rather less sensitive to polarization and gave less fluctuation in ν-OTDRs. In our experiments, the SNSPD was biased at 0.85 of its critical current, where the bias current was low enough to avoid afterpulses. Thus, compared to ν-OTDRs using gated APDs, the ν-OTDRs using SNSPDs showed an absence of fluctuations from afterpulses and charge subsistence^6^.

From the measured traces, we found that the only significant source for fluctuation in our ν-OTDR system was the statistical noise due to the quantum nature of light and the binary photon-counting mechanism. As shown in Fig. 5a, the fluctuation clearly increases as measurement time decreases. Therefore, there is a tradeoff between the fluctuation and measurement time. As the photon detection can be taken as a Poisson distribution, the fluctuation (*δ*) normalized to the average photon count (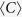) is 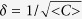, where 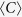 is a function of measurement time (*t*_m_).


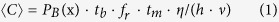


In Eq.1, *P*_B_(*x*) is the power of the backscattered light at location *x*, *t*_b_ is the window size of each sampling bin, *f*_r_ is the repetition rate of the incident pulses, *η* is the detection efficiency, *h* is the Planck constant, and ν is the frequency of the incident light.

We extracted the OTDR traces from 175 km to 215 km to analyze their fluctuations. A linear fitting was applied to get the average count <*C*>, from which the variation (*C*-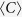)/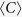 at each time bin was calculated. We calculated the 10^th^ percentile (*C*_10_) and the 90^th^ percentile (*C*_90_) based on a Poissonian distribution with an intensity of 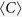 and converted them to the upper boundary (*C*_90_-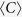)/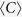 and lower boundary (*C*_10_-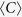)/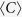 of the fluctuation for each figure. As shown in Fig. 5c-e, variations at each time bin were well confined between the two boundaries, showing that the intrinsic statistical noise dominated the fluctuations in our SNSPD-based ν-OTDR.

## Conclusions

We have demonstrated a ν-OTDR with a dynamic range of 46.9 dB at a two-point resolution of 0.1 km in a total measurement time of 40 minutes. These long-haul and high-resolution features were caused by using an SNSPD which had high detection efficiency, low dark counts, high counting rate, and operated in free-running mode. The measurement time was fast: the time for measuring the fiber tail after 100 km was reduced to one minute. The two-point resolution was reduced to 20 m with a dynamic range of 39.8 dB by launching 100 ns wide optical pulses. In this work, the cryogenic system included a liquid helium cryostat and a dip-stick setup, which was convenient and low cost in laboratories but lacked of high reliability. Thus, we are also working on designing a cryogenic system using a Gifford-McMahon refrigerator to sustain a temperature of 2.5 K for multiple SNSPDs. In this way, the ν-OTDR system will be able to operate continuously and reliably outside of the laboratory. Because of the longer sensing distance and better resolution, our long-haul OTDR is promising in applications where strict requirements are needed, such as monitoring long submarine fibers or precisely fixing positions of fiber breakpoints in bridges or metros. The technology to operate SNSPD at high photon flux is also useful in other applications, such as time-of-flight measurement, and fluorescence imaging^20^,^21^.

## Additional Information

**How to cite this article**: Zhao, Q. *et al.* Long-haul and high-resolution optical time domain reflectometry using superconducting nanowire single-photon detectors. *Sci. Rep.* **5**, 10441; doi: 10.1038/srep10441 (2015).

## Supplementary Material

Supplementary Information
