# Supplementary material for: Long-haul and high-resolution optical time domain reflectometry using superconducting nanowire single-photon detectors
Source: Sci Rep. 2015 May 28;5:10441. doi: 10.1038/srep10441 (PMC4447072; doi:10.1038/srep10441)
Supplement: Supplementary Information [file srep10441-s1.doc]

Long-haul and high-resolution optical time domain reflectometry using superconducting nanowire single-photon detectors

Qingyuan Zhao^1^, Lan Xia^2^, Chao Wan^1^, Junhui Hu^3^, Tao Jia^1^, Min Gu^1^, Labao Zhang^1^, Lin Kang^1^, Jian Chen*^1^, Xuping Zhang^2^ and Peiheng Wu^1^

^1^Research Institute of Superconductor Electronics (RISE), School of Electronic Science and Engineering, Nanjing University, 22 Hankou Road, Nanjing 210093, China.

^2^Institute of Optical Communication Engineering, School of Management and Engineering, Nanjing University, 22 Hankou Road, Nanjing, 210093, China

^3^College of Physics Science and Technology, Guangxi Normal University, 15 Yucai Road, Guilin, 541004, China

E-mail: [chenj63@nju.edu.cn](mailto:chenj63@nju.edu.cn)

Supplementary materials

1. Noise correction

We did a correction to remove the noise from dark counts to linearize the OTDR trace at the fiber end. The probability to have a count due to photon detection is given by

*p*_S_ = 1-e^-^*^μ^*^∙^*^η^* (1)

where *μ* is the average photon number in each time bin. The measured counts can be either from photon detections or dark counts. In each time bin *t*_b_, the overall probability (*p*_C_) is equal to *C*/(*t*_m_∙*f*_r_), where *C* is the total counts, *t*_m_ is the measurement time and *f*_r_ is the repetition rate of optical pulses. *p*_C_ is given by

*p*_C_ = 1-(1-*p*_D_)∙e^-^*^μ^*^∙^*^η^* (2)

where the dark count probability is given by *p*_D_ = *D*_K_∙*t*_b_.


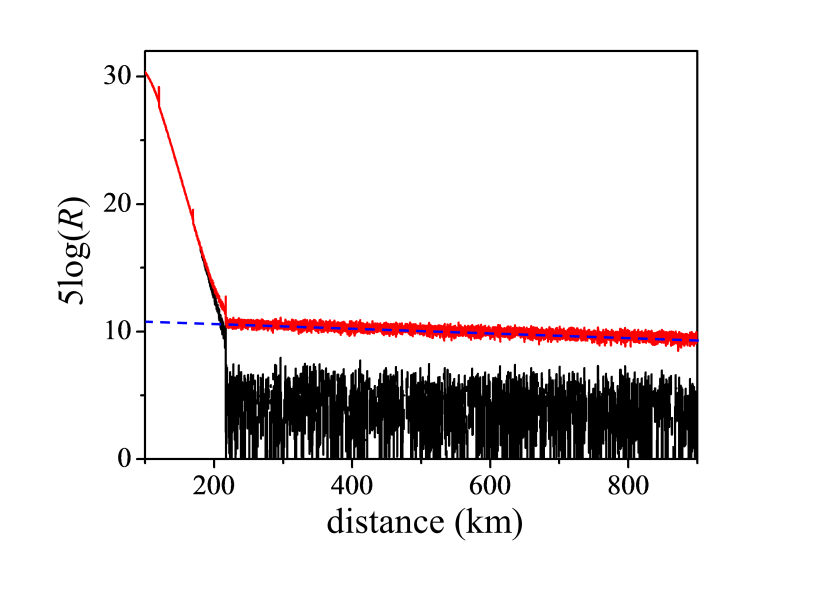


Figure S1. OTDR traces taken for 30 minutes with (black) and without noise-correction (red). The blue-dashed curve is the fitting curve for the noise floor which varies over time/distance. After noise-correction, as shown in the black curve, the noise floor is almost flat and the fiber tail shows a linear decay.

In these measurements, dark counts or spurious counts were not only from the SNSPD but also related to the OTDR measurement. As shown in figure S1, after the fiber end, the noise floor was not flat but with a decay over time. We thought the increased noise floor came from the overlapped backscattered signals generated from the multiple Fresnel reflections of the input optical pulses at the fiber connections. To estimate the distance(*x*)-varying noise, we did an exponential fitting of the noise floor and extrapolated the noise before the fiber end. The fitting expressions for OTDR traces at different measurement time were:

$D_{\text{K1}}=\text{ 161}.1\cdot\text{e}^{-8.027\times10^{-4}x}$, for *t*_m_ = 1800s (3)

$D_{\text{K2}}=\text{ }50.04\cdot\text{e}^{-7.993\times10^{-4}x}$, for *t*_m_ = 600s (4)

$D_{\text{K3}}=\text{ }5.014\cdot\text{e}^{-7.986\times10^{-4}x}$, for *t*_m_ = 60s (5)

Solving *μ* from equation (1) and equation (2), the mean number of photon per gate is expressed as

$\mu\text{=}\frac{1}{\eta}\cdot\ln(\frac{1-p_{\text{D}}}{1-p_{\text{C}}})$ (6)

Therefore, by substituting *p*_D_, we could have *p*_S_. The total counts from photon detections is then *R* = *p*_S_∙*t*_m_∙*f*_r_.

2. Fitting SNSPD’s counting rate versus light power

To calibrate the saturated ν-OTDR trace, it is necessary to find an analytical or numerical expression for the counting rate (*CR*) to the input power (*P*_in_). Here, we give the method for fitting the measured dynamic range of the SNSPD in the main article.

However, it is difficult to find an analytical expression for the SNSPD, since the recovery of *η* has an exponential recovery profile and detections interact between each other. Using the same method that we have discussed in our previous work, a numerical solution can be found. We set *p*(0) as 1 at time 0, and calculate *p*(*t*_n_) in the following time bins with a time interval of Δ*t*. Because every detection at time *t*_m_ switches the detector and starts a new recovery of detection efficiency, *p*(*t*_n_) can be written into the superposition of all possible preceding detections with the weighted efficiency *η*(*t*_n_-*t*_m_). The profile of *η*(*t*) is acquired experimentally at a low *λ* where photon detections are isolated. Considering dark counts, we have a numerical expression for *p*(*t*_n_) by

$p(t_{\text{n}})=p(t_{\text{n-1}})\cdot[1-\overline{p_{\text{S}}}(\Delta t)\cdot\overline{p_{\text{D}}}]+\sum_{m=0}^{n-2} p(t_{\text{m}})\cdot[\prod_{i=m\text{+}1}^{n-1} \overline{p_{\text{S}}}(t_{i}-t_{\text{m}})\cdot\overline{p_{\text{D}}}]\cdot[1-\overline{p_{\text{S}}}(t_{\text{n}}-t_{\text{m}})\cdot\overline{p_{\text{D}}}]$ (7)

where $\overline{p_{\text{S}}}(t)=e^{-\eta(t)\cdot\lambda\cdot\Delta t}$ is the probabilities of no photon detection at time *t* in a time window of Δ*t* and $\overline{p_{\text{D}}}=1-D_{\text{K}}\cdot\Delta t$ is the probabilities of no dark count in a time window of Δ*t*. For a high *λ*, the time-varying detection efficiency *η*(*t*_n_) = *p*(*t*_n_)/(*λ*∙Δ*t*) oscillates and finally stays at a constant baseline which is taken as the average detection efficiency <*η*>. Thus, we have the solution for *CR*, which is

*CR* = <*η*>∙*λ*  (8)

3. Fitting dynamic range versus measurement time

We define the power of the backscattered signal at the beginning of the fiber as *P*_B0_ and the minimal detectable power as *P*_m_ in one time bin. Thus, the dynamic range is given by

*dynR* = 5∙log(*P*_B0_/*P*_m_) (9)

We derive *P*_B0_ from the OTDR trace from the first step measurement in Fig. 3a. First, we calculate the mean photon number (*μ*_x_) at location *x* where the incident photons is weak and the SNSPD operates in single-photon counting, therefore, there is *μ*_x_ ≈ *C*(x)/(*t*_m_∙*f*_r_∙*η*), where *C*(x) is the counts at *x* and *t*_m_ is the measurement time, and *P*_B_(x) = *μ*_x_∙h∙ν/*t*_b_, where *P*_B_(x) is the backscattered signal power at *x* and h∙ν is the photon energy. For example, we set *x* = 170 km, and calculated *μ*_x_ = 0.26 and *P*_B_(170km) = -104.9 dBm. Then, we extrapolated *P*_B0_ from *P*_B_(x)considering the total round-trip loss of the 2*x* long fiber with an experimental fiber attenuation of 0.19 dB/km and the 1.4 dB loss from the five fiber connections. After this calculation, we had *P*_B0_ = -38.9 dBm.

*P*_m_ is defined as the minimal detectable power that gives photon detections equal to the noise or fluctuation from dark counts in each time bin. Dark counts follow a Poissonian distribution, thus the fluctuation is $\Delta N_{\text{D}}=\sqrt{D_{\text{K}}\cdot t_{\text{b}}\cdot f_{\text{r}}\cdot t_{\text{m}}}$, where *D*_K_ is the dark count rate. Therefore, we have *P*_m_ from the following equation

$P_{\text{m}}\text{=}\frac{h\nu}{\eta}\sqrt{\frac{D_{\text{K}}}{t_{\text{b}}\cdot f_{\text{r}}\cdot t_{\text{m}}}}$ (10)

After *P*_B0_ and *P*_m_ are known, the dynamic range *dynR* can be solved as a function of the measurement time. We used equation (1) to fit Fig.5(a) in the main article by setting *D*_K_ as 260 Hz (Dark counts in the ν-OTDR measurement were higher than the value in characterizing SNSPD.
